# Machine learning methods for the study of cybersickness: a systematic review

**DOI:** 10.1186/s40708-022-00172-6

**Published:** 2022-10-09

**Authors:** Alexander Hui Xiang Yang, Nikola Kasabov, Yusuf Ozgur Cakmak

**Affiliations:** 1 Department of Anatomy, University of Otago, Dunedin, New Zealand; 2 KEDRI, School of Engineering, Computer and Mathematical Sciences, Auckland University of Technology, Auckland, New Zealand; 3 George Moore Chair of Data Analytics, Ulster University, Londonderry, UK; 4 ABI, The University of Auckland, Auckland, New Zealand; 5 Medtech Core NZ, Auckland, New Zealand; 6 Brain Health Research Centre, Dunedin, New Zealand; 7 Centre for Health Systems and Technology, Dunedin, New Zealand; 8 Centre for Bioengineering and Nanotechnology, Point-of-Care Technologies Theme, University of Otago, Dunedin, New Zealand

**Keywords:** Cybersickness, Detection, Prediction, Biometrics, Physiological, Review, Systematic, Machine learning, AI, Neural networks, Virtual reality, Extended reality, Simulator

## Abstract

This systematic review offers a world-first critical analysis of machine learning methods and systems, along with future directions for the study of cybersickness induced by virtual reality (VR). VR is becoming increasingly popular and is an important part of current advances in human training, therapies, entertainment, and access to the metaverse. Usage of this technology is limited by cybersickness, a common debilitating condition experienced upon VR immersion. Cybersickness is accompanied by a mix of symptoms including nausea, dizziness, fatigue and oculomotor disturbances. Machine learning can be used to identify cybersickness and is a step towards overcoming these physiological limitations. Practical implementation of this is possible with optimised data collection from wearable devices and appropriate algorithms that incorporate advanced machine learning approaches. The present systematic review focuses on 26 selected studies. These concern machine learning of biometric and neuro-physiological signals obtained from wearable devices for the automatic identification of cybersickness. The methods, data processing and machine learning architecture, as well as suggestions for future exploration on detection and prediction of cybersickness are explored. A wide range of immersion environments, participant activity, features and machine learning architectures were identified. Although models for cybersickness detection have been developed, literature still lacks a model for the prediction of first-instance events. Future research is pointed towards goal-oriented data selection and labelling, as well as the use of brain-inspired spiking neural network models to achieve better accuracy and understanding of complex spatio-temporal brain processes related to cybersickness.

## Introduction

Cybersickness is a type of visually induced motion sickness experienced in virtual environments [1]. It is well-recognized that the symptoms of nausea, dizziness, fatigue and oculomotor problems have been a barrier to mainstream adoption of VR technology [1]. The utility of VR includes not just gaming and entertainment [2], but applications for professional training in healthcare, aerospace, industrial, defence, disaster safety and emergency procedures [3]. VR can also be used for planning cost-effective architectural designs [4]. Moreover, there is potential for VR well-being applications in high-stress reduction [5], exposure therapy to reduce anxiety and trauma [6, 7] and health-related interventions using VR neuromodulation [8]. The importance of VR is further highlighted in environments with restricted social interactions such as those imposed by the recent COVID-19 pandemic, allowing people to connect despite physical boundaries [9]. Clearly, the world is progressing towards an integrated metaverse, which embraces immersive mixed realities [10]. It is, therefore, crucial to tackle the issue of cybersickness.

While visible physiological signs can be tell tales of an ongoing cybersickness event, it is still a perceptual disorder that is not always apparent until it is communicated [11]. Machine learning provides a way to log cybersickness events without overreliance on communication from VR users. This information enables timely prevention and treatment. The source of which comes from widely available sensor technology, which allows features to be derived from biometric and physiological data.

Measures include:

- Electrical activity in the brain [12–27]
- Electrical potentials of eye movement [25, 28, 29]
- Heart rate and heart rate variability [28, 30, 31]
- Gastric activity [25, 29]
- Muscle activity [24]
- Respiration rate [25, 28–32]
- Skin conductivity [28–31, 33]
- Eye blinks [25, 28, 29]
- Body movements [25, 34, 35]

Recently, Yildirim [36] reviewed four studies that used deep learning of electroencephalogram (EEG) data for the classification of cybersickness. The studies presented promising results, with accuracies in the range of 83.33–99.12% [36]. The review noted that extra care should be taken to report EEG data transformations, or lack thereof, as part of pre-processing. In addition, it was recommended that studies report clear descriptions for deep learning architectures, such as tensor shape, number and type of layers, activation functions and hyperparameters.

Moving forward, several other aspects in the wider literature require clarification. Across studies, there is a lack of homogeneity with regard to the definitions of detection and prediction. It is important to distinguish between the two to be clear about the extent of a machine learning model’s capability. In this review, we define detection as the identification of data related to an ongoing cybersickness event. In contrast, we define prediction as the forecasting of future cybersickness using data prior to the event.

From a clinical perspective, the course of action against cybersickness would differ depending on the ability to detect or predict it. A model that detects cybersickness provides an opportunity for timely intervention, whereas a model that predicts potentially allows for early prevention.

A machine learning model’s applicability is heavily dependent upon the data fed into it. Therefore, further investigation into the experimental design, method of data selection and data labelling in studies is needed. In addition, subject demographics, immersion environment and participant activity all influence the data obtained and the features extracted; ultimately framing the context of a model’s results. An overview of these items across published studies is required, particularly to help future studies have clear goal implementation when designing an experiment or machine learning architecture.

Finally, cybersickness is a result of dynamic spatio-temporal processes in the brain, involving different spatially located areas over time [37, 38]. Appropriate machine learning (ML) methods can help provide a better understanding of these processes, at both the group and individual level, but this has not been clearly assessed yet in previous studies.

The goal of this systematic review was to analyse relevant studies pertaining to various physiological and biometric-based machine learning approaches towards the detection and prediction of cybersickness. The review provides a discussion of the experimental methods for data collection, processing and machine learning analysis within different architectures. In addition, suggestions for future exploration are discussed.

The research questions for this review are as follows:

1. How have studies been able to detect or predict cybersickness?
2. What stimulus type, environment and participant activity contributed towards cybersickness induction?
3. How are the reviewed articles comparable?
4. Which features are the most important?
5. What new information about brain activities related to cybersickness have been revealed by the studies?

### Contributions

In summary, according to the research questions, we contribute the following:

- An in-depth summary of study design and details for each reviewed study
- A differentiation between prediction and detection studies
- Awareness of the consequences of mislabeling cybersickness data
- A highlight of the most informative features for machine learning and classification, with the caution they may not be generalizable or interpretable.
- Considerations for practical translation of machine learning algorithms to wearable devices for consumer usage, including number and type of sensors for different use cases.
- Future suggestions for machine learning of physiological data related to cybersickness.

Our justification for choosing these research questions and items of discussion, stems from the need to develop study protocols that properly capture cybersickness data according to specific research goals. The aspects chosen influence the type of data processed and are crucial to the meaning of machine learning outputs. Furthermore, this affects not just the practical translatability of machine learning algorithms, but also the explainability of models that pave the way for the generation of new knowledge about cybersickness. Deliberation on these aspects will not only help to develop better study designs, but also aid in human adaptation to digital environments.

## Methods

### Database search

PubMed and IEEE Xplore databases were used to cover the intersection between biomedical and life sciences literature with that of computer science and engineering. Google Scholar was used to manually screen papers from an extensive array of journals and conferences based on their titles and abstracts. The review was written with PRISMA guidelines [39] for systematic reviews in mind. Eligible publications needed to utilize a stimulus to induce cybersickness through a virtual visual medium, such as either a screen projection, desktop display, VR head-mounted device or simulator environment. Publications also needed to apply machine learning on physiological data or physical measures of body and eye movement for the classification of cybersickness data samples. We define a study to be an instance of machine learning even if it solely uses regression analysis. Studies were excluded if an analysis was applied for knowledge discovery about physiological correlations with cybersickness without any event detection, prediction or estimation of sickness levels. Studies analyzing only non-physiological data such as VR content or subjective questionnaire scores were excluded. Other exclusions were reviews, methodological articles, conference abstracts, and publications, where full-text was not available through our institutions. See Table 1 for the database search and selection criteria.

**Table 1:** Database search and selection criteria

Database search	
Electronic database	1. Pubmed 2. Google Scholar 3. IEEE Xplore
Inclusion criteria	1. Articles that develop or validate a prediction or detection model using any data source, e.g., individual patient data or from electronic records 2. Studies must utilize a stimulus with a virtual visual medium 3. Any machine learning analysis and physiological processing or physical measures of body/eye movement collected from wearable devices for the classification of cybersickness 4. All outcome measures in any format, e.g., continuous, binary, ordinal, multinomial, time-to-event
Exclusion criteria	1. Studies using machine learning to classify only non-physiological data, e.g., VR content or questionnaire scores 2. Studies that only investigate physiological correlations with cybersickness as a form of knowledge discovery 3. Reviews, Concept papers and abstracts 4. Full text not available

This review included all studies from 2001 to 10th November 2021. The search strategy was adapted from PICO criteria (Patient or population, Intervention, Control or Comparison, Outcome and Study; Table 2).

**Table 2:** Search strategy

Search strategy	
Population	Studies using physiological data to build cybersickness classification algorithms
Intervention	Inducement of cybersickness to create labelled data for classification
Comparison	Different models and their utility for clinical intervention
Outcome	Ability to detect or predict cybersickness
Study type	Quantitative study
Keywords	Cybersickness OR visually induced motion sickness OR simulator sickness AND physiological AND machine learning AND virtual reality

### Search terms

Cybersickness OR visually induced motion sickness OR simulator sickness AND physiological AND machine learning AND Virtual Reality.

### Screening process

Titles and abstracts of potential studies were assessed independently by three reviewers (AHX Yang, N. Kasabov, YO. Cakmak), according to the Preferred Reporting Items for Systematic Reviews and Meta-Analysis (PRISMA) guidelines [40]. The studies that met the inclusion and exclusion criteria were discussed and evaluated based on their content relative to the five research questions outlined above. Two reviewers (AHX Yang, YO. Cakmak) evaluated the full-text studies independently, while the third reviewer (N. Kasabov) resolved disagreements.

### Data extraction and analysis

An initial risk of bias (ROB) assessment was run, from a conservative viewpoint. The ROB covered four domains from the PROBAST recommendations each containing their own signalling question items to judge risk of bias [41].

Responses were formulated as yes (Y) or probably yes (PY) for the absence of bias and no (N), probably no (PN), or no information (NI) to indicate a potential for bias. Overall judgement of risk of bias for publications was defined as high, low, or unclear. Although it is important to note that bias in itself is not necessarily a criticism of the choice of study design, which could be scientifically reasoned, but an assessment of erroneous assumptions that may lead to misleading conclusions based on machine learning results.

Additional data items were extracted to answer the five research questions outlined above which are specific to the field of cybersickness classification. The data items include subject demographics, immersion type, participant activity, information on different machine learning models, reporting styles, data segment labelling, preprocessing methods, biometric and neurophysiological features relevant to cybersickness and EEG specifications. These data items are listed in Tables 3, 4, 5, 6, 7, 8, and 9, which sort studies by year from earliest to most recent. Two reviewers (AHX Yang, YO. Cakmak) independently extracted and assessed the data from the included studies, while the remaining reviewer (N. Kasabov) cross-referenced, clarified differences in interpretation, and then confirmed a standardized response. Any disagreements reached a consensus and were resolved by the third reviewer (N. Kasabov) after discussion.

**Table 3:** Subject demographic including sample size, gender, age range and mean with standard deviation where available

Author	*N*	Male	Female	Age range	Mean
*Subject demographics*					
Nam et al. [12]	45	25	20	18–26	21.9
Yu et al. [13]	7	–	–	21–24	–
Wei et al. [16]	6	–	–	–	–
Wei et al. [14]	6	–	–	–	–
Ko et al. [15]	10	–	–	–	–
Lin et al. [17]	10	–	–	–	–
Ko et al. [18]	6	–	–	–	–
Lin et al. [19]	17	–	–	–	–
Dennison et al. [29]	20 (9 completed)	14	6	–	–
Pane et al. [26]	9	6	3	25–35	–
Mawalid et al. [21]	9	7	2	–	–
Khoirunnisaa et al. [20]	9	7	2	–	25.1
Dennison et al. [25]	20	15	5	> 18	–
Wang et al. [34]	11	7	4	–	25.83 ± 4.58
Garcia-Agundez et al. [28]	66	–	–	–	–
Jeong et al. [22]	24	13	12	20–33	–
Li et al. [35]	20	20	0	18–27	22.8
Kim et al. [42]	202	–	–	–	–
Liao et al. [27]	130	65	65	6–23	–
Li et al. [23]	18 (6 excluded)	19	5	–	29.3
Lee, Alamaniotis [43]	31	29	2	–	24.04 ± 2.75
Islam et al. [30]	31 (8 excluded) = 23	29	2	–	24.04 ± 2.75
Islam et al. [31]	31 (8 excluded) = 23	29	2	–	24.04 ± 2.75
Martin et al. [33]	103	86	17	–	26.12 ± 6.31
Recenti et al. [24]	28	22	6	–	23.8 ± 1.2
Oh, Kim [32]	20 (2 excluded) = 18	8	12	–	–

Dashes (–) are put, where information was missing or not available

**Table 4:** Immersion type including mode of stimulus, VR content, platform usage, and participant activity

Author	Mode of stimulus	VR content	Platform (moving/still)	Standing/sitting/active
*Immersion type and participant activity*				
Nam et al. [12]	3D virtual environment simulator	Virtual background of buildings	None	Unclear
Yu et al. [13]	360 degree Simulator, 6 degrees freedom motion platform	Auto driving	Moving, sync with simulator	Passive sitting (visual + vestibular)
Wei et al. [16]	360 degree Simulator, 6 degrees freedom motion platform	Auto driving	Moving, sync with simulator	Passive sitting (visual + vestibular)
Wei et al. [14]	360 degree Simulator, 6 degrees freedom motion platform	Auto driving	Moving, sync with simulator	Passive sitting (visual + vestibular)
Ko et al. [15]	360 degree Simulator, 6 degrees freedom motion platform	Auto driving	Moving, sync with simulator	Passive sitting (visual + vestibular)
Lin et al. [17]	360 degree Simulator, 6 degrees freedom motion platform	Auto driving	Moving, sync with simulator	Passive sitting (visual + vestibular)
Ko et al. [18]	360 degree Simulator, 6 degrees freedom motion platform	Auto driving	Moving, sync with simulator	Passive sitting (visual + vestibular)
Lin et al. [19]	360 degree Simulator, 6 degrees freedom motion platform	Auto driving	Moving, sync with simulator	Passive sitting (visual + vestibular)
Dennison et al. [29]	Display Monitor 1920 × 1280 resolution, Oculus Rift	VR exploration	None	Passive sitting (visual)
Pane et al. [26]	47 Inches LED Monitor HD-1366 × 768 resolution	Mirrors edge	None	Sitting (active playing, visual)
Mawalid et al. [21]	47 Inches LED Monitor HD-1366 × 768 resolution	Mirrors edge	None	Sitting (active playing, visual)
Khoirunnisaa et al. [20]	47 Inches LED Monitor HD-1366 × 768 resolution	Mirrors edge	None	Sitting (active playing, visual)
Dennison et al. [25]	Oculus rift DK2	VR exploration	None	Standing (visual)
Wang et al. [34]	HTC Vive HMD	Virtual exploration	None	Standing (visual)
Garcia-Agundez et al. [28]	Oculus rift DK2	VR plane flying	None	Active sitting (visual)
Jeong et al. [22]	FOVE VR headset	6 VR videos	None	Unclear
Li et al. [35]	Projected screen	Forward/backward video, auto driving	None	Standing (visual)
Kim et al. [42]	HTC vive HMD	44 VR videos	None	Unclear
Liao et al. [27]	HTC vive HMD	Roller coaster, space simulator, boat	None	Passive siting (visual)
Li et al. [23]	HTC vive HMD	VR roaming	None	Passive sitting (visual)
Lee and Alamaniotis [43]	HTC vive HMD	VR rollercoaster	None	Passive sitting (visual)
Islam et al. [30]	HTC vive HMD	VR rollercoaster	None	Passive sitting (visual)
Islam et al. [31]	HTC vive HMD	VR rollercoaster	None	Passive sitting (visual)
Martin et al. [33]	Oculus rift	Multiple VR games	None	Active sitting (visual)
Recenti et al. [24]	VR Goggles HMD + moving platform	Open sea boat on waves	Moving, sync with VR waves	Standing in all stages, active balancing (visual + vestibular)
Oh and Kim [32]	HTC vive HMD	VR rollercoaster	None	Passive sitting (visual)

**Table 5:** Biosignal recordings, machine learning algorithms, performance and type of classification system in terms of detection or prediction

Authors	Biosignal	Algorithm	Binary/multiclass	Accuracies	Classification type
*Machine learning models*					
Nam et al. [12]	EEG, EOG, ECG, finger tip skin temperature, PPG, skin conductance	ANN, 2-layer feedforward neural network	Binary	Minimum mean square error 0.092	Detection
Yu et al. [13]	EEG	GMLC, KNN, SVM	Binary	KNN, NWFE 99.9%	Detection
Wei et al. [16]	EEG	RBFNN, SVR, LR	Multi class motion sickness level	84.07% LR 84.75% RBFNN, 86.92% SVR	Detection
Wei et al. [14]	EEG	RBFNN	Multi class motion sickness level	84.39% ± 0.75	Detection
Ko et al. [15]	EEG	LR, PCR	Multi class motion sickness level	PCR 78.3% ± 8.0 LR 64.7% ± 15.6	Detection
Lin et al. [17]	EEG	SVM	Multi class motion sickness level	36.3–73.3%	Detection
Ko et al. [18]	EEG	SVM	Multi class motion sickness level	58.5–97.0%	Detection
Lin et al. [19]	EEG	SONFIN, LR, SVR	Multi class motion sickness level	Broad band EEG SONFIN 82% ± 2 SVR 79% ± 3 LR 80% ± 3	Detection
Dennison et al. [29]	ECG, EGG, EOG, blink rate, PPG, breathing rate, GSR	Stepwise regression	SSQ score estimation	Adjusted *R*^2^: Cybersickness 0.296 Nausea 0.101 Oculomotor 0.674 Disorientation 0.268	Detection
Pane et al. [26]	EEG	CN2 rule induction algorithm, decision tree, SVM	Multiclass	CN2 88.9% Decision tree 72.2% SVM 83.3%	Detection
Mawalid et al. [21]	EEG	Naïve Bayes, KNN	Binary	KNN 83.3% Naïve bayes 88.9%	Detection
Khoirunnisaa et al. [20]	EEG	SVM-RBF, KNN, LDA	Binary	SVM-RBF 83.3% KNN 83.0% LDA 100%	Detection
Dennison et al. [25]	EEG, ECG, EOG, blink rate, breathing rate, EGG, postural sway, head movement	LDA, KNN, Naive Bayes, decision tree, ADABoostM2, and bagged decision trees	Multiclass	Unimodal Feature Bag classifier: EEG: 93.80% Posture: 83.48% Breathing rate: 81.32% HMD sensors: 78.40% Avatar movement: 74.40% ECG: 68.44% EOG: 61.84% EGG: 48.52% Multimodal feature fusion: Bag: 95% KNN: 93% ADABoost: 92%	Detection
Wang et al. [34]	Postural sway	LSTM	SSQ score estimation	Pearson correlation coefficient *r* = 0.89 between SSQ score and loss (measure of difference in postural sway between pre and post VR exposure)	Detection
Garcia-Agundez et al. [28]	ECG, EOG, blink rate, breathing rate, GSR	Fine Gaussian SVM, linear SVM, KNN	Binary and Multiclass	Binary: fine Gaussian SVM: no cs: 57.6%, minor: 74.2%, severe: 81.8% Ternary: KNN: 58%	Detection
Jeong et al. [22]	EEG	DNN, CNN	Binary cutoff	DNN 98.02% CNN 98.82%	Detection
Li et al. [35]	EEG, postural sway, head body movement	KNN, LR, RF, MLP in a voting classifier	Multiclass	single subject binary classification: 91.1% multiple subject binary classification: 76.3% 3 class classification: 86.7% Severe, 50.4% moderate, 79.1% mild, 68.9% average accuracy	Detection
Kim et al. [42]	EEG	CNN, LSTM, RNN	Multiclass	LSTM with EEG 87.13% ± 1.51 Combined LSTM EEG + CNN-RNN visual features: 89.16% ± 1.87% visual predictor alone: 79.03 ± 1.24%	Detection
Liao et al. [27]	EEG	LSTM, SVM, MLP, CNN	Binary cutoff custom sickness index	1 min: 83.94%, 5-min 83.33%, 10 min 83.92% 82.83% for RNN-LSTM, CNN at 73.13%. MLP at 71.31% and LibSVM at 62.58%	Prediction
Li et al. [23]	EEG	KNN, polynomial-SVM, RBF-SVM	Binary	Single subject binary classification: polynomial-SVM 92.83%, KNN 90.97%, RBF-SVM 90.74% Multiple subject classification: 79.25%, 77.5%, 73.84%, respectively	Detection
Lee and Alamaniotis [43]	EEG	DESOM with auto encoder for clustering, KNN	Binary	DESOM Purity index 96.87%	Prediction
Islam et al. [30]	ECG, breathing rate, GSR	LSTM regression analysis	Multiclass	MAE 8.7%	Prediction
Islam et al. [31]	ECG, breathing rate, GSR	CNN-LSTM	Multiclass based on sickness score estimation	Detection: 97.44%, Prediction: 87.38%	Detection and Prediction
Martin et al. [33]	BVP, EDA	SVM, GB, RF, LR	Sickness rating estimation, Binary and Multiclass	Model trained on all participants: LR: R^2^ 0.75 RF: binary 91.7%, multiclass 86.2% One model for each participant: LR: *R*^2^ 0.40 RF: binary 89%, multiclass 85.9%	Prediction
Recenti et al. [24]	EEG, EMG, heart rate	RF, GB tree, SVM, KNN, MLP	Binary	RF: IPV 75.9%, INM, 79.5%, IMS 74.7%	Detection
Oh and Kim [32]	BVP, respiratory signal	DELM with SVM, KNN, RF, ADAboost stacked into CNN	Multiclass	SVM: 94.23%, KNN: 92.44%, RF: 93.20%, ADABoost: 90.33%, DELM: 96.48%	Detection

For simplicity, relevant top accuracies/results are reported. Artificial neural network (ANN) gaussian maximum likelihood classifier (GMLC), k-nearest neighbour (KNN), support vector machine (SVM), radial basis function neural network (RBFNN), support vector regression (SVR), linear regression (LR), principal component regression (PCR), self-organizing neural fuzzy inference network (SONFIN), linear discriminant analysis (LDA), long short-term memory (LSTM), Deep neural network (DNN), convolutional neural network (CNN), multilayer perceptron (MLP), deep embedded self-organizing map (DESOM), random forest (RF), deep ensemble learning model (DELM) IPV (physiological index), INM (neurological/muscle strain index), IMS (motion sickness index), mean absolute error (MAE), non-parametric weighted feature extraction (NFWE)

**Table 6:** Reporting styles and data labelling

Author	Biosignal	Report	Non-cybersickness labelling	Cybersickness labelling
Nam et al. [12]	EEG, EOG, ECG, finger tip skin temperature, PPG, skin conductance	Verbal	Data points not labelled as cybersick	Within 3 s of report while immersed
Yu et al. [13]	EEG	Joystick scale	Participant defined time segments	Continuous scale
Wei et al. [16]	EEG	Joystick scale	Participant defined time segments	Continuous scale
Wei et al. [14]	EEG	Joystick scale	Participant defined time segments	Continuous scale
Ko et al. [15]	EEG	Joystick scale	Participant defined time segments	Continuous scale
Lin et al. [17]	EEG	Joystick scale	Participant defined time segments	Continuous scale
Ko et al. [18]	EEG	Joystick scale	Participant defined time segments	Middle of motionsickness level graph and after highest sickness rating
Lin et al. [19]	EEG	Joystick scale	Participant defined time segments	Continuous scale
Dennison et al. [29]	ECG, EGG, EOG, blink rate, PPG, breathing rate, GSR	SSQ	N/A (SSQ score estimation)	Entire VR immersion
Pane et al. [26]	EEG	SSQ cut-off score	Before gameplay	Tailend of gameplay
Mawalid et al. [21]	EEG	SSQ cut-off score	Before gameplay	tailend of gameplay
Khoirunnisaa et al. [20]	EEG	SSQ cut-off score	Before gameplay	total gameplay
Dennison et al. [25]	EEG, ECG, EOG, blink rate, breathing rate, EGG, postural sway, head movement	In game input via controller	Score of zero for 'no symptoms' on a zero to three point scale	30 s intervals
Wang et al. [34]	Postural sway	SSQ	N/A (SSQ score estimation)	N/A (SSQ score classification)
Garcia-Agundez et al. [28]	ECG, EOG, blink rate, breathing rate, GSR	SSQ	SSQ score cut off	Entire VR immersion
Jeong et al. [22]	EEG	Keyboard marker	Unclear	entire video
Li et al. [35]	EEG, postural sway, head body movement	Keyboard marker	During VR, before video movement	Varying interval throughout video
Kim et al. [42]	EEG	Likert scale	Video contents scored '1: comfortable' on Likert-like scale	Mid video
Liao et al. [27]	EEG	Verbal	Lack of cybersickness report during VR immersion	Report of sickness, entire recording
Li et al. [23]	EEG	Tact switch	Before VR immersion	Varying interval throughout immersion
Lee and Alamaniotis [43]	EEG	Mouse click	During VR, before video movement	2 s timespan, 1 s before cybersickness report
Islam et al. [30]	ECG, breathing rate, GSR	Verbal	Sickness scale cutoff for entire VR immersion	Sickness scale cutoff for entire VR immersion
Islam et al. [31]	ECG, breathing rate, GSR	Verbal	Before VR immersion, and before video movement	Entire VR immersion
Martin et al. [33]	BVP, EDA	Verbal	Score of zero on VR sickness scale	Window sizes of 10, 30, 60, 90, 120 s before report of sickness with a score equal to or more than 1
Recenti et al. [24]	EEG, EMG, heart rate	MSSQ	N/A (index classification)	N/A
Oh and Kim [32]	BVP, respiratory signal	Verbal	no report of cybersickness and pre-immersion neutral states	Entire VR immersion

Simulator sickness questionnaire (SSQ), motion sickness questionnaire (MSSQ)

**Table 7:** Preprocessing methods

Author	Biosignal	Preprocessing
*Preprocessing methods*		
Nam et al. [12]	EEG, EOG, ECG, finger tip skin temperature, PPG, skin conductance	Power band extraction, standard deviation of EOG, mean R–R of ECG, mean and standard deviation of fingertip skin temperature, PPG and skin conductivity. Data segments for all variables calculated in period 3 (30 s after to the end of VR immersion) ratioed to period 1 and 2 (1 min before VR immersion and 30 s after)
Yu et al. [13]	EEG	1–50 Hz high and low pass filter, 250 Hz down sampling, ICA, component clustering, FFT and conversion to decibel power
Wei et al. [16]	EEG	1–50 Hz high and low pass filter, 250 Hz down sampling, ICA, component clustering, FFT and conversion to decibel power
Wei et al. [14]	EEG	1–50 Hz high and low pass filter, 250 Hz down sampling, ICA, component clustering, FFT and conversion to decibel power
Ko et al. [15]	EEG	1–50 Hz high and low pass filter, 250 Hz down sampling, ICA, component clustering, FFT and conversion to decibel power
Lin et al. [17]	EEG	1–50 Hz high and low pass filter, 250 Hz down sampling, ICA, component clustering, FFT for PSD and subsequent conversion to decibel power
Ko et al. [18]	EEG	1–50 Hz high and low pass filter, 250 Hz down sampling, ICA, component clustering, FFT for PSD and conversion to decibel power
Lin et al. [19]	EEG	1–50 Hz high and low pass filter, 250 Hz down sampling, ICA, component clustering, FFT for PSD and conversion to decibel power
Dennison et al. [29]	ECG, EGG, EOG, blink rate, PPG, breathing rate, GSR	ECG bandpass filter 0.5–30 Hz, EGG bandpass filter 0.005–2 Hz and FFT with Hamming window, percentage band power for tachygastric and bradygastric activity, respiration bandpass filter 0.1–1 Hz, PPG bandpass filter 0.1–10 Hz, EOG bandpass filter 0.1–5 Hz, baseline normalization for skin conductivity, standard deviation of yaw, pitch and roll head rotation in degrees
Pane et al. [26]	EEG	FIR bandpass 1–40 Hz, ICA, ratio logarithmic of PSD (percentage power), change in percentage power pre-stimuli to post stimuli (percentage change) Daubechies 4 wavelet (db4) function
Mawalid et al. [21]	EEG	ICA, Chebyshev bandpass filter type II
Khoirunnisaa et al. [20]	EEG	FIR bandpass 1–40 Hz, ICA, Discrete Wavelet transform, Welch's method for PSD
Dennison et al. [25]	EEG, ECG, EOG, blink rate, breathing rate, EGG, postural sway, head movement	ECG bandpassfilter 0.5–30 Hz, EEG bandpass filter 0.1–30 Hz, data interpolation from other channels after manual artifact removal, ICA, FFT, EOG bandpass filter 0.1–5 Hz, EGG bandpass filter 0.005–2 Hz, FFT with Hamming window, percentage band power for tachygastric and bradygastric activity, respiration bandpass filter 0.1–1 Hz, standard deviation of yaw, pitch and roll rotation degrees, average and standard deviations in weight changes for postural sway. Any missing data replaced and standardized across features
Wang et al. [34]	Postural sway	–
Garcia-Agundez et al. [28]	ECG, EOG, blink rate, breathing rate, GSR	Mean and standard deviation on game content vectors
Jeong et al. [22]	EEG	4–45 Hz automatic filter. Data sets created based on 4 custom signal quality weightings, min max normalization/standardization
Li et al. [35]	EEG, postural sway, head body movement	Channel integration, paired interception, simultaneous artifact removal, FFT for PSD
Kim et al. [42]	EEG	Bandpass filter 0.3–100 Hz, notch filter at 60 Hz, FFT applied through a sliding Hann window. EEG Data transformed into a 8 channel stacked spectogram
Liao et al. [27]	EEG	FFT for PSD
Li et al. [23]	EEG	Elliptical pass band filter 0.5–30 Hz, Fourier transform, 7 level WPT
Lee and Alamaniotis [43]	EEG	256 Hz down sampling
Islam et al. [30]	ECG, breathing rate, GSR	*z*-score removal of outliers
Islam et al. [31]	ECG, breathing rate, GSR	*z*-score removal of outliers, 1 Hz down sampling, min–max normalization
Martin et al. [33]	BVP, EDA	BVP inter-beat interval extraction bandpass filter 0.66–3.33 Hz, frequency and time domain feature computation, EDA tonic and phasic computation
Recenti et al. [24]	EEG, EMG, heart rate	0.1–40 Hz high pass and low pass filter, 300 microvolts upper limit, common average reference, interpolation for removed channels, baseline correction, DC offset correction, Welch's method for PSD, relative power averaged across all channels
Oh and Kim [32]	BVP, respiratory signal	Exclusions of data samples after inspection for artifacts

Independent component analysis (ICA), fast Fourier transform (FFT), power spectral density (PSD), wavelet packet transform (WPT), direct current (DC)

**Table 8:** Feature extraction, selection methods, fusion with other biosignals for machine learning and the important features from each study

Author	Biosignal	Feature extraction/selection methods	Feature fusion	Important features
*Biometric and neurophysiological features relevant to cybersickness*				
Nam et al. [12]	EEG, EOG, ECG, finger tip skin temperature, PPG, skin conductance	PCA	Yes	Fz, Cz, Pz, O1, O2, theta (5–8 Hz), alpha (9–13 Hz), beta (14–30 Hz), gamma (31–50 Hz), standard deviation of EOG, mean R-R of ECG, mean and standard deviation of fingertip skin temperature, PPG and skin conductivity
Yu et al. [13]	EEG	PCA, LDA, NWFE, FFS/BFS for PSD	None	Delta (0.1–3 Hz), theta (4–7 Hz), alpha (8–13 Hz), beta (14–30 Hz)
Wei et al. [16]	EEG	PCA for PSD	None	Broadband frequency 1–50 Hz
Wei et al. [14]	EEG	Genetic algorithm for PSD	None	Broad band frequencies, especially delta (1–3 Hz), alpha (8–12 Hz), beta (13–30 Hz), channels unknown
Ko et al. [15]	EEG	PCA for PSD	None	Fp1, Fp2, C3, C4, Pz, Oz
Lin et al. [17]	EEG	Inheritable bi-objective combinatorial genetic algorithm (IBCGA) for PSD	None	Gamma band (21–50 Hz) (parietal area and occipital midline)
Ko et al. [18]	EEG	Extended inheritable bi-objective combinatorial genetic algorithm (e-IBCGA) for PSD	None	Beta (13–20 Hz) and gamma (21–30 Hz) (parietal area and occipital midline)
Lin et al. [19]	EEG	PCA for PSD	None	Alpha (8–12 Hz) and gamma (21–30 Hz) combined, broad band signals (occipital midline)
Dennison et al. [29]	ECG, EGG, EOG, blink rate, PPG, breathing rate, GSR	Pearson correlation with SSQ cut-off	None	Bradygastric (less than 2 cycles of contraction per minute) percentage power, mean blinks, mean breaths, MSSQA
Pane et al. [26]	EEG	ANOVA to rank frequency band feature importance based on 3 class labels (none, low, high cybersickness)	None	Decrease of Percentage power of beta (12–30 Hz) in O1
Mawalid et al. [21]	EEG	Mean, variation, standard deviation, number of peak and ratio logarithmic of power spectral density (power percentage)	Yes	Alpha (8–13 Hz) and beta (13–20 Hz) combined for all 14 channels, as well as their variation and standard deviation
Khoirunnisaa et al. [20]	EEG	Channel selection through information gain and correlation-based on feature selection	None	Power percentage beta (16–32 Hz) for F3 > 01 > 02 > F4 > AF4
Dennison et al. [25]	EEG, ECG, EOG, blink rate, breathing rate, EGG, postural sway, head movement	Greedy sequential forward feature selection process	Yes	Number of breaths per 30 s, number of blinks per 30 s, heart rate, ECG R-peak amplitude, avatar right-left displacement, % of slow wave stomach activity (less than 2 cycles of contraction per minute), 13 EEG powerband features (0.1–30 Hz) (left frontal alpha, left motor theta, left parietal beta, left occipital delta, left occipital theta, left occipital alpha, right frontal theta, right frontal gamma, right motor delta, right motor theta, right parietal beta, right parietal delta, and right occipital gamma)
Wang et al. [34]	Postural sway	LSTM encoder to learn features	No	Reconstruction error of postural sway signal
Garcia-Agundez et al. [28]	ECG, EOG, blink rate, breathing rate, GSR	HR, breathing rate, respiration rate using peak detection algorithm	Yes	Combination of game content vectors, heart rate, blink rate, respiratory rate, galvanic skin response
Jeong et al. [22]	EEG	Raw data + power bands	Yes	Signal quality weightings
Li et al. [35]	EEG, postural sway, head body movement	PCA for Power band, centre of pressure, head and waist movement	Yes	Combination of theta (4–8 Hz) and alpha (8–13 Hz) in all 31 channels, center of pressure, head and waist movement
Kim et al. [42]	EEG	Temporal and spectral networks	Yes	P3, P4
Liao et al. [27]	EEG	PSD	Yes	Broadband frequencies, 0–100 + Hz
Li et al. [23]	EEG	Combined 4 rhythm energy ratios for all channels	None	FP1, FP2, C3, C4, P3, P4, O1, O2
Lee and Alamaniotis [43]	EEG	EEGNET to capture features	None	Unknown
Islam et al. [30]	ECG, breathing rate, GSR	Pearson-correlation coefficient analysis, min, max, running average for HR, HRV and GSR	Yes	Min, max, running average for heart rate, heart rate variability and galvanic skin response
Islam et al. [31]	ECG, breathing rate, GSR	Pearson-correlation coefficient analysis, min, max, running average for HR, HRV and GSR	Yes	Min, max, running average for heart rate, heart rate variability and galvanic skin response
Martin et al. [33]	BVP, EDA	HRV time domain and frequency domain computation, EDA tonic and phasic feature computation	Yes	Binary and multiclassification Rank 1/50: Baseline EDA minimum amplitude Binary classification only Rank 5/50: Heart rate Multiclassification only Rank 5/50: pNN50
Recenti et al. [24]	EEG, EMG, heart rate	Power spectra based on previous studies	Yes	Beta EEG signals (13–35 Hz), EMG at right gastrocnemius 40–132 Hz, average HR
Oh and Kim [32]	BVP, respiratory signal	Manual selection of HRV and respiratory signal features	Yes	HR, HRV amplitude, LF, HF, and LF/HF ratio, respiratory rate and respiratory value

Principle component analysis (PCA), linear discriminant analysis (LDA), non-parametric weighted feature extraction (NFWE), forward feature selection (FFS), backward feature selection (BFS), power spectral density (PSD), simulator sickness questionnaire (SSQ), long short-term memory (LSTM), heart rate (HR), heart rate variability (HRV), galvanic skin response (GSR), electrodermal activity (EDA), low frequency (LF), high frequency (HF).

**Table 9:** EEG devices, channels and powerband frequencies

Authors	Device	Channels used	**Power band frequencies**
*EEG specifications*			
Nam et al. [12]	Five channel 200 Hz sampling rate	Five channel EEG Fz, Cz, Pz, O1, O2	Theta (5–8 Hz), alpha (9–13 Hz), beta (14–30 Hz), gamma (31–50 Hz)
Yu et al. [13]	Unknown, 32 channel	P8, T8, CP6, FC6, F8, F4, C4, P4, AF4, Fp2, Fp1, AF3, Fz, FC2, Cz, CP2, PO3, O1, Oz, O2, PO4, Pz, CP1, FC1, P3, C3, F3, F7, FC5, CP5, T7, P7	Delta (0.1–3 Hz), theta (4–7 Hz), alpha (8–13 Hz), beta (14–30 Hz)
Wei et al. [16]	NuAmps 32 channel 500 Hz sampling	P8, T8, CP6, FC6, F8, F4, C4, P4, AF4, Fp2, Fp1, AF3, Fz, FC2, Cz, CP2, PO3, O1, Oz, O2, PO4, Pz, CP1, FC1, P3, C3, F3, F7, FC5, CP5, T7, P7	1–50 Hz (undefined)
Wei et al. [14]	NuAmps 32 channel 500 Hz sampling	P8, T8, CP6, FC6, F8, F4, C4, P4, AF4, Fp2, Fp1, AF3, Fz, FC2, Cz, CP2, PO3, O1, Oz, O2, PO4, Pz, CP1, FC1, P3, C3, F3, F7, FC5, CP5, T7, P7	Delta (1–3 Hz), theta (4–7 Hz), alpha (8–12 Hz), beta (13–30 Hz), gamma (31–50 Hz)
Ko et al. [15]	NuAmps 32 channel 500 Hz sampling	FP1, FP2, C3, C4, Pz, Oz	1–60 Hz
Lin et al. [17]	NuAmps 32 channel 500 Hz sampling	P8, T8, CP6, FC6, F8, F4, C4, P4, AF4, Fp2, Fp1, AF3, Fz, FC2, Cz, CP2, PO3, O1, Oz, O2, PO4, Pz, CP1, FC1, P3, C3, F3, F7, FC5, CP5, T7, P7	Delta (0.1–3 Hz), theta (4–7 Hz), alpha (8–12 Hz), beta (13–20 Hz), gamma (21–50 Hz)
Ko et al. [18]	NuAmps 32 channel 500 Hz sampling	P8, T8, CP6, FC6, F8, F4, C4, P4, AF4, Fp2, Fp1, AF3, Fz, FC2, Cz, CP2, PO3, O1, Oz, O2, PO4, Pz, CP1, FC1, P3, C3, F3, F7, FC5, CP5, T7, P7	Delta (0.1–3 Hz), theta (4–7 Hz), alpha (8–12 Hz), beta (13–20 Hz), gamma (21–30 Hz)
Lin et al. [19]	NuAmps 32 channel 500 Hz sampling	P8, T8, CP6, FC6, F8, F4, C4, P4, AF4, Fp2, Fp1, AF3, Fz, FC2, Cz, CP2, PO3, O1, Oz, O2, PO4, Pz, CP1, FC1, P3, C3, F3, F7, FC5, CP5, T7, P7	Delta (0.1–3 Hz), theta (4–7 Hz), alpha (8–12 Hz), beta (13–20 Hz), gamma (21–30 Hz)
Pane et al. [26]	Emotiv Epoc + 14 channel 10/20 system 256 Hz	O1, O2	Theta (4–8 Hz), alpha (8–12 Hz), beta (12–30 Hz)
Mawalid et al. [21]	Emotive Epoc + 14 10/20 system 256 Hz	AF3, F7, F3, FC5, T7, P7, O1, O2, P8, T8, FC6, F4, F8, AF4	Alpha (8–13 Hz), beta (13–20 Hz)
Khoirunnisaa et al. [20]	Emotiv Epoc + 14 channel 10/20 system 256 Hz	AF3, F7, F3, FC5, T7, P7, O1, O2, P8, T8, FC6, F4, F8, AF4	Theta (4–8 Hz), alpha (8–16 Hz), and beta (16–32 Hz)
Dennison et al. [25]	Advanced Neuro Technologies 64 channel cap	All 64 with some removal and interpolation due to artifacts	Delta, theta, alpha, beta, and gamma bands (0.1–30 Hz), undefined power band ranges
Jeong et al. [22]	Emotiv Epoc + 14 channel 10/20 system 2048 Hz downsampled upon export to 128 Hz	AF3, F7, F3, FC5, T7, P7, O1, O2, P8, T8, FC6, F4, F8, AF4	Theta (4–8 Hz), alpha (8–12 Hz), low beta (12–16 Hz), high beta (16–25 Hz), gamma (25–45 Hz)
Li et al. [35]	Neuroscan SynAmps2 Model 8050 Eeg amplifier and data acquisition system. 64 channel 1000 Hz AC sampling	FP1, FP2, AF3, AF4, F3, F4, F7, F8, FZ, FC1, FC2, FC5, FC6, C3, C4, Cz, CP1, CP2, CP5, CP6, P3, P4, P6, P7, P8, Pz, PO3, PO4, O1, O2, Oz	Theta (4–8 Hz), alpha (8–13 Hz)
Kim et al. [42]	Eight channel 250 Hz, 16 bits	Unclear	none
Liao et al. [27]	Neuroskymind wave mobile 512 Hz	FP1	Broad band frequencies: delta, theta, low alpha, high alpha, low beta, high beta, low gamma, high gamma
Li et al. [23]	Opebci 256 Hz sampling 8 channel Ag/AgCl dry electrode brain cap	FP1, FP2, C3, C4, P3, P4, O1, O2	Delta (0.5–3 Hz), theta (4–7 Hz), alpha (8–13 Hz), beta (14–30 Hz)
Lee and Alamaniotis [43]	Cognionics 10/20 system 32 channels, sometimes 64 channels was used, 256 or 512 Hz, respectively	Not reported	raw EEG
Recenti et al. [24]	AntNeuro 64-channel dry electrode cap 500 Hz	All 64 with some removal and interpolation due to artifacts	Delta (0.5–4 Hz), theta (4–8 Hz), alpha (8–13 Hz), beta (13–35 Hz), low gamma (35–40 Hz)

## Results

### Search and Selection

**Fig. 1:**
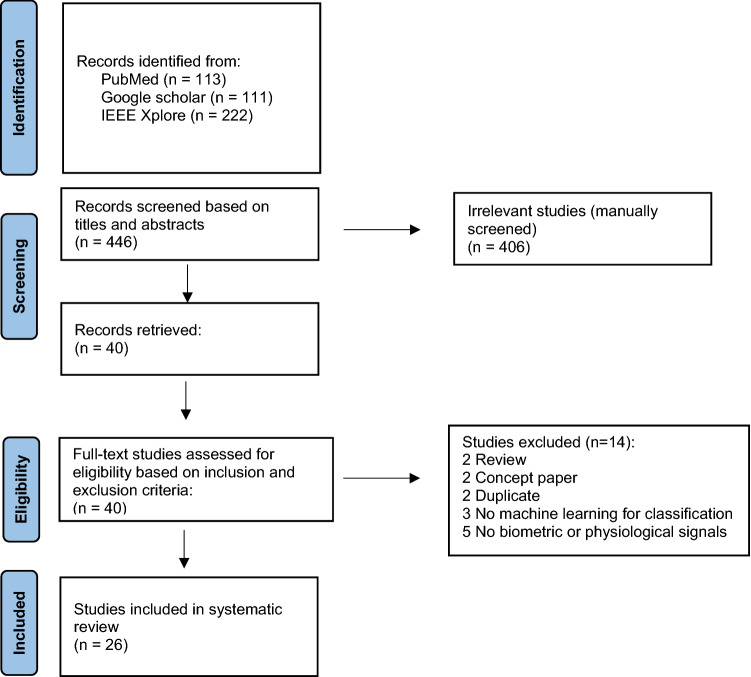
Flowchart for study screening and selection process

The database search identified 446 studies. After manual screening for titles and abstracts, 40 studies remained based on the inclusion/exclusion criteria. 14 studies were further excluded upon assessment of the full text. Among them, there were 2 reviews, 2 concept papers, 2 duplicates, 3 did not use machine learning for classification and 5 did not analyze biometric or physiological signals. The remaining 26 papers were included in this review. Figure 1 presents a flowchart of the study screening and selection process.

### Risk of Bias

All studies had an overall low risk of bias (see Table 10). Of these, one study [27] had a “No” for item 17, because data segments were labelled as cybersick that may have contained control data. Another study [23] sampled control data that may have been influenced by conditions other than the lack of cybersickness, such as non-VR immersion. One study [12] had “No” for item 14, because only participants who felt nausea were used and “NI” for 15 and 19, because it was unclear if there were missing data or if the model accounted for overfitting. Most studies (21/26), had a low participant sample size (item 12). Two studies [15, 17] had a “No” for items 3 and 5 as they employed feature selection algorithms that differed among participants. Eleven studies (see Table 10) were either unclear, or did not report exclusion of participants with health disorders (item 2) which could affect the feature variables used in classification of cybersickness and even contribute to already having nausea, although this was not weighted as heavily in the overall ROB assessment.

### Subject demographics

The sample size of subjects ranges from 6 to 202 throughout the 26 studies. Of the 26 studies, 17 report gender, and 9 do not. Most of the studies consist of predominantly male participants, while one study uses comparatively more females [32] and a few balance both male and female participants [12, 22, 27]. With regard to age reporting, 15 out of 26 studies report ages, of which 12 studies either report the age range or mean with standard deviation. For details, refer to Table 3.

### Immersion type and participant activity

Both vestibular and visual stimuli or visual alone have been used. Visual mediums include 360° simulators, LED desktop display monitors, projected screens and head mounted displays (HMDS), refer to Table 4. Moving platforms have been incorporated, including ones that mimic sea waves on a ship [24] and automatic driving simulators [13–19], both of which introduce vestibular stimuli in synchronicity with their virtual environments. Studies have made participants play games [20, 21, 26, 28, 33], undergo virtual navigation [12, 23, 25, 34], or watch virtual videos [22, 27, 30–32, 35, 42, 43]. During these activities, there would doubtless exist some self-induced vestibular stimuli through head movements or balancing while standing. It is unclear in three studies whether a standing or sitting posture was used during either virtual simulator environment immersion or VR HMD usage while watching videos [12, 22, 42]. Each study’s mode of stimulus, VR content, platform usage and type of participant activity are summarized in Table 4.

### Machine learning models

Table 5 summarizes the machine learning analysis undertaken by all 26 studies. This includes biosignal recordings, algorithm(s) used, classification types in terms of binary, multiclass, or score estimation, accuracies, and abilities of models to detect or predict cybersickness. Out of all the studies, five have built predictive algorithms, while the rest have detected cybersickness [30, 27, 31, 33, 43].

### Biosignals recorded

Studies have utilized electroencephalogram (EEG), electrooculography (EOG), electrocardiogram (ECG), photoplethysmography (PPG), electrogastrogram (EGG), electromyogram (EMG), Respiration signals, galvanic skin response (GSR) also known as electrodermal activity (EDA), eye tracker, postural sway and body sensors at the head and waist. Derived variables include power band analysis of EEG [12–27], electrical potentials of eye movement from EOG [25, 28, 29], heart rate and heart rate variability measures from ECG [25, 28–33] which can also be calculated from blood volume pulse (BVP) obtained from PPG [32, 33], gastric activity from EGG [25, 29], muscle activity from EMG [24], respiration rate [25, 28–32], skin conductivity from GSR [28–31, 33], eye blinks [25, 28], weight shifts [25, 34] and center of pressure for postural sway [35], and head and waist movements from body sensors [25, 35].

### Neural networks

Nam et al. [12] is the earliest known publication in the field of automatic detection of cybersickness. The study used a 2-layer feed-forward artificial neural network to partially detect nausea timings. Other studies used deep neural networks (DNN) [22], including multilayer perceptron (MLP) [35], radial basis function neural network (RBFNN) [14, 16], convolutional neural network (CNN) [42], recurrent neural network-long short term memory (RNN-LSTM) [27], as well as self-organizing neural fuzzy inference network (SONFIN) [19], and deep embedded self-organizing map (DESOM) with a CNN auto encoder and decoder from EEGnet [43].

DNNs are neural networks with two or more fully connected hidden layers, usually stacked linearly in groups. MLP and RBFNN are DNNs that differ in how their outputs are determined. MLP networks work globally and their outputs are decided by all neurons. In contrast, RBFNNs are local approximation networks whose outputs are determined by hidden units in local receptive fields. CNNs are also a type of DNN. They learn patterns in the data through filtering in convolutional layers, then pass data through pooling layers to compress the size of representation, allowing for parameters to be computed faster. CNNs are optimized for image data. RNNs are another type of DNN. They learn representations in an iterative manner, using outputs of a layer as recurrent inputs to the same or other layers. LSTMs are a subset of RNNs, that allow for the learning and reconstruction of signals, and allow for the prediction of future signals based on previous timesteps of data [27, 30, 31, 42]. SONFIN and DESOM are examples of self-organizing neural networks, that work based on weights between nodes.

### Other ML tools

There are a variety of other non-neural network machine learning tools employed in the reviewed studies. These include: maximum gaussian likelihood estimator [13]; a simple tool that uses a gaussian distribution, where maximum probability of a label occurs if the data points are closer to their mean value, support vector machines (SVM) which try and find the best hyperplane between data sets that belong to different classes [13, 18, 17], its variations with kernel functions of polynomial SVM [23], SVM-radial basis function (RBF) [20, 23], support vector regression (SVR) [16, 19], linear regression (LR) which assumes a linear relationship between input and output variables [15, 16, 19, 33], linear discriminant analysis (LDA) which finds linear combinations of features that can separate classes [20, 25], principle component regression (PCR) [15] based on principle component analysis, naïve bayes based on probability theorem [21], *k*-nearest neighbours (kNN) [13, 20, 21, 23, 25, 28, 32, 35, 43] which labels new data according to the majority of nearby pre-labelled data, decision trees that employ a flowchart-like structure for decision making [25, 26], including random forest [24, 35] and bagged decision tree which reduces the variance of a decision tree [25], gradient boosting trees to minimize errors [24], and CN2 rule induction that extracts rules from features in a data set [26]. Studies that have used these machine learning tools have detected but not predicted future cybersickness states.

### Classification types

Some studies use a mix of binary and multiclass classification. Binary classification refers to the labelling of two different classes in a machine learning task, whereas multiclass classification refers to multiple labels, which in this review concerns levels of cybersickness severity. Other studies use machine learning to estimate simulator sickness questionnaire (SSQ) scores, which is another form of multiclassification.

### Data selection

A summary of reporting styles, cybersickness and non-cybersickness data labelling is summarized in Table 6.

Multiple methods for choosing data segments related to cybersickness have been used. These include a report on the first instance of cybersickness perception [12, 43]. Commonly, entire video segments or VR immersion sessions were used if they had been labelled as cybersickness [20, 22, 27–32]. Specific timeframes were also picked. This is so that either cybersickness is highest or most likely at the selected data segment [18, 21, 26] to capture cybersickness intervals [23, 25, 35], or to predict future states using past data sectioned in various temporal window sizes [33].

The method of labelling data segments as ‘non-cybersickness’ varied among studies. Some studies choose to label ‘non-cybersickness’ as the beginning data segment while wearing an HMD, with a static image and no camera movement in the VR environment [35, 43]. Others have opted to use data segments recorded before VR immersion or gameplay [20, 21, 23, 26, 31, 32]. Studies have also chosen to select data that does not meet different cutoffs using SSQs scores or rating scales [25, 28, 30, 33, 42]. Alternatively, chosen data samples were those with no report of cybersickness during VR immersion [27, 32], or data segments not corresponding to the cybersickness label [12]. In studies, where motion sickness levels were estimated, the ‘non-cybersickness’ labels and time segments were participant defined via a self-operated joystick scale, keeping note that the earliest timepoint of recording in these studies already included a moving video [13–19]. Several studies included in this review do not label data specifically as ‘non-cybersickness’. These consist of two studies that attempt to estimate SSQ scores [29, 34], while one tries to classify EEG, heart rate and EMG signals into specific binary indexes related to motion sickness [24]. Thus, the classification of ‘non-cybersickness’ data refers to participants in many different environments across studies, in terms of pre and during immersion states as well as exposure to movement and non-movement of visual scenes.

### Preprocessing

Preprocessing refers to the manipulation of raw data so that specific variables can be generated for further processing. During preprocessing, methods are used to reduce redundancy and extract meaningful data. Methods include down sampling to reduce the amount of data, filters to remove artifacts and select only a portion of data, manual artifact correction, computational artifact correction, such as *z*-scores and independent component analysis (ICA), weighting data based on signal quality, data transformation using variations of Fast Fourier Transform (FFT), and optimization of fixed windowed time segments. This is summarized in Table 7.

### Features extraction, selection and fusion

Feature extraction and selection is a method to get a subset of relevant features/variables from the data, to be fed as an input into a machine learning tool. The idea is to reduce complexity and feed algorithms with the most relevant data. Studies have focused on spatial locations of EEG channels, as well as temporal, frequency and amplitude variables from recorded biosignals. Methods used range from manual selection to statistical computations, genetic algorithms, and convolutional networks. Most notably, three studies focus on selecting as few EEG channels as possible, showing that one to three channels can be used for cybersickness detection [20, 26, 27]. In addition to obtaining features, feature fusion allows data from multiple biometric and physiological signals to be combined for classification. Out of 26 studies, 13 studies have fused features from multiple signals. Feature extraction and selection techniques and details of studies that have applied feature fusion are displayed in Table 8.

### Important features for classification

Important features (Table 8) were included according to the following criteria: correlation to cybersickness scores or by optimal classification accuracies, appearance in multiple machine learning models [25], and if eight or fewer channels are involved and named [12, 15, 23]. In cases, where the authors have not explicitly pointed out or done an analysis to rank the importance of features, all the features used in the classification are included in the list for transparency of information and comparison to other studies [12, 13, 16, 27, 32]. For a full compilation of EEG devices, channels and power band frequencies used in the reviewed studies, refer to Table 9. Frequency ranges for each power band are reported in Table 9 because of the inconsistencies between studies, especially in regard to differentiation of the beta and gamma bands.

Across reviewed studies, the overall importance of broad band EEG frequency signals is revealed; with a focus on alpha (8–12 Hz), beta (13–20 Hz) and gamma bands (21–30 Hz) in channels relating to cortical regions in occipital areas (O1, O2, Oz) [12, 15, 17–20, 23, 25, 26], parietal areas (P3, P4, Pz) [12, 15, 17, 18, 25, 42] and left and right frontal areas [15, 20, 23, 25].

Postural sway [24, 34, 35], head and body movement [25, 35] and blink rate [25, 28, 29] have shown to be useful features for cybersickness classification. Among others, heart rate has consistently been an important feature across studies [24, 25, 28, 30–33]. A heart rate variability (HRV) indicator, pNN50, which is the percentage of N–N intervals within 50 ms, has also been a high contributor to results obtained in both binary and multiclassification tasks [33]. Alongside ECG derived variables, respiration rate has been identified as an important feature for cybersickness classification and estimation [25, 28, 32]. Electrodermal activity also known as galvanic skin response has shown promise as an important feature in four studies [28, 30, 31, 33], but did not make a statistically significant correlation with SSQ scores in another [29].

## Discussion

Overall, the type of population, and an assessment of algorithm utility remains ambiguous and wanting. The wide differences in study protocols, data labelling and processing make it difficult to compare the reviewed studies. Although scarce, the available studies have highlighted that it is possible to track cybersickness using a variety of biosignals. The robustness of these signals to noise in practical settings, however, requires careful consideration. Looking forward, the discovery of new information about cybersickness requires machine learning tools that are open and explainable. These aspects are discussed below.

### Subject demographics

#### The subject demographics are biased towards males, with a lack of age range reporting

There is evidence that females tend to be more susceptible to visually-induced motion sickness than males [45]. Both vestibular and visual motion sickness incidence in females tends to be higher than in males, but with no difference in the severity of symptoms [46–49]. Emerging evidence suggests that interpupillary distance non-fit, while wearing HMDs is one driving factor for this gender discrepancy [50]. A meta-analysis of factors associated with cybersickness suggested that age may be a contributor to likelihood of sickness [51]. As much as possible, the gender demographic and age range should be reported as a means of identifying possible influences towards study results. Studies may want to balance or separate analyses of females and males. An argument could be made, however, that female and male analysis should be done together so that a machine learning model can be subject to a wide population and subject demographic.

### Prediction versus detection

#### Studies have presented evidence for the detection of cybersickness and further cybersickness events, but predictive capabilities for novel, first-instance cybersickness events are in question

It is not necessarily the machine learning algorithm used that determines the model’s ability to detect or predict cybersickness but the choice of data segments. Data segments in prediction studies have been chosen before the onset of cybersickness, allowing machine learning models to be trained on data prior to the future event. Training on prior data is inbuilt for studies using LSTMs, allowing researchers to specify timesteps of certain lengths as training for signal reconstruction of relative future timepoints [52]. However, studies do not specify the timing of cybersickness occurrence in each data sample, meaning that crossing a certain timepoint, the model could be trained on data already related to cybersickness to detect future cybersickness events. This leads to the question: are the models truly predictors, detectors, or a combination of both? If indeed they are a combination, these models could be defined as a detection-based prediction model, in which current cybersick data can be used to predict future cybersick data. With the exception of Islam et al. [31], the literature lacks models only trained on clear non-cybersickness segments that are used to detect future cybersick events. Furthermore, literature so far has focused on detecting cybersickness events using data segments related to VR immersion, but few have tried to predict future cybersick events using pre-VR immersion baselines [31]. To the best of our knowledge, there is a lack of machine learning studies focused on predicting susceptibility or future cybersick events in VR using the normal resting physiological state of an individual.

### Labelling

#### Labelling cybersick data in long windows could increase false positives and negatives

A drawback to labelling entire video segments or VR immersion sessions based on a post SSQ or on a first instance reporting basis is the lack of temporal precision. Large portions of data could in truth represent the wrong label, increasing rates of false positives and false negatives during classification. Where possible, a solution would be to have data segments that are in relatively small temporal windows, ideally near the time of reporting and to avoid large temporal windows in the order of minutes.

#### Non-cybersick data should be labelled under the same experimental conditions as cybersick data

If ‘non-cybersickness’ data is not under the same conditions as ‘cybersick’ labelled data, the risk increases that a machine learning algorithm learns the difference between conditions influenced by a different environment, rather than the perception of cybersickness itself.

### The reviewed studies are difficult to compare

Stimulus type, environment and participant activity differ greatly across studies. Different stimuli inputs, as well as virtual environments and scene content, and standing and sitting conditions make studies difficult to compare. With regard to comparing EEG studies, vestibular stimuli and visual stimuli used to induce motion sickness activates a vast array of different cortical areas in the brain [37, 53–62]. Previous research has also found that certain movements of visual scenes inside VR can alter HRV [8, 63]. Constant clockwise rotation of the visual environment has been found to inhibit cardiac parasympathetic activity [8]. Root mean square of successive differences of R-R intervals (RMSSD) and standard deviation of R-R intervals (SDRR), measures of parasympathetic activity, were also found to be decreased in forward visual movement compared to backward visual movement during VR immersion in a rollercoaster ride [63]. In addition, vestibular stimuli is known to increase cardiac sympathetic activity [64], meaning that different activity levels could potentially alter HRV variables and associated feature importance. Thus, on top of feature extraction and selection methods, extra care needs to be taken when deciding which features to choose for a new study.

### Features

#### Parietal, occipital and frontal cortical areas in the alpha, beta and gamma band are highly related to cybersickness in machine learning studies

Gamma band signal importance for classification is in line with recent findings, showing increased gamma power with increased cybersickness severity [65]. In the application of frequency filters to reduce artifacts and noise, one should be careful not to filter out valuable information that could exist past chosen frequency boundaries.

It is also important to keep in mind that evidence for parietal and occipital feature importance in cybersickness stem mainly from two studies that either have considerably more vestibular stimuli through moving platforms or use desktop LED displays rather than VR HMDs [23, 25], and are thus different in environment and stimulation type. More research is needed as a cross reference to determine if these features are common regardless of environment or stimulation type.

Nevertheless, the involvement of multiple brain regions suggests that interactions and connections exist across different spatial locations. In an fMRI study by Toschi et al. [37], reduced connectivity was found in nausea states compared to baseline, between the right and left primary visual cortex (V1), as well as increased connectivity between the right middle temporal visual area (MT + /V5) and anterior insular, and between the left MT + /V5 and the middle cingulate cortex. Thus, deeper brain structures could be involved that may not necessarily be revealed in the above machine learning EEG studies.

#### None of the machine learning methods used so far have clearly revealed the complex, dynamic, spatio-temporal processes in the brain related to cybersickness

Studies have relied on statistical comparisons between sets of cybersickness and normal/control data, to find important frequency-based power spectra in isolated channels, or spatial clusters over large brain regions. Beyond this, communication between spatial locations, functional networks, and temporally relevant information still needs to be explored. More complex machine learning algorithms have not been employed for knowledge extraction. This important task requires new machine learning methods on its own.

#### Heart rate, heart rate variability, postural sway, head and body movement, blink rate, breathing rate and EDA are informative features

Important features of increased heart rate have been found to be correlated with increasing cybersickness severity [65]. Heart rate variability measures other than pNN50, like changes in the average duration of N–N intervals (AVGNN), and changes in the standard deviation of N–N intervals (STDNN) have been correlated with SSQ scores [66]. Head and body movement could potentially exacerbate sensory mismatches while in VR and is part of the generally accepted sensory mismatch theory [67]. The contribution of increased postural sway to detect cybersickness is rooted in the postural instability theory for motion sickness [68], although a study has shown a weak link between postural instability and cybersickness. Eye movements have also been hypothesized to generate motion sickness [69]. Blink rate seems likely a symptom of oculomotor disturbances, which is a subcategory for simulator sickness [70]. Breathing rate appears to be an important feature under experimental conditions [25, 28, 29, 32]. Furthermore, controlled diaphragmatic breathing has also been studied to manage cybersickness through modulation of the parasympathetic nervous system [71].

#### The fewer channels, the better

The number of channels is important for simplicity and ergonomic reasons. The fewer the channels, the less bulky an automated cybersickness detection or prediction system device needs to be. Using 64 channels [24, 25] is unwieldy outside of lab conditions in an operational or consumer setting. It also requires good signal quality from most, if not all channels. Therefore, it is suggested that studies investigate channel reduction methods while still preserving accuracy.

#### Features discovered from extraction and selection, or those that contribute the most to optimal classification accuracies, may not be generalizable or interpretable

While studies have drawn attention to many features, it is important to note that the most correlated features may not be the most frequently chosen by feature extraction/selection tools [14]. The generalizability of features to different experimental settings still needs to be assessed. This will help build a robust model of physiological activity during cybersickness events. Features related to cybersickness can also be statistically tested and compared to their importance in machine learning models. This can be a helpful indicator of their correlation versus importance. Finally, features obtained for novel, first-instance future cybersickness event prediction are predictive features. They may not necessarily be the same features occurring during cybersickness. Thus, caution is warranted as the same features listed in this review may not be generalizable to a study attempting to predict cybersickness from pre-immersion baselines.

#### Some forms of signal acquisition are more practical than others

VR content has been explored as a data source for cybersickness classification as well [42, 72]. Current interventions include narrowing the field of view or slightly changing the visual scene to attenuate cybersickness symptoms [73]. While virtual environment contents provide useful data in retrospect, they cannot be the medium for intervention in all situations. Such would be the case for high-stakes training simulations or even immersive real-time operations that require large fields of view and a high degree of realism. Unfortunately, even with live streaming, the predictive nature of virtual content would be limited without availability beforehand. VR content is a stimulus and cannot be used to directly assess individual cybersickness. Biometric and physiological signals, on the other hand, can measure human states and responses to both the environment and clinical treatment [74, 75]. Moreover, wearable devices are portable and can still be used while engaging with VR in a non-sedentary manner. This level of practicality and freedom is currently not possible with other forms of data capture such as with the brain (magnetoencephalography [76], functional magnetic resonance imaging [77]). Still, not all signals from wearable devices are as practical as each other. EEG, EOG, ECG, PPG, EGG and eye tracker could be used in combination for cybersickness detection and prediction in a practical consumer or operational setting. Respiration signals, GSR, postural sway and body movement, in contrast, could be difficult to implement. In high activity settings, these signals may become riddled with artifact and noise (from heavy erratic breathing, profuse sweating, and large amounts of movement), reducing the practicality of these signals.

### Future suggestions

Along with reporting of subject demographics, proper labelling of data and investigations of channel reduction methods to reduce the number of sensors needed to capture this data, there are several other future suggestions. The future of machine learning on cybersickness involves not just the alteration of visual displays for human ergonomic comfort, but the understanding of physiological states and subsequent mitigation of potentially harmful perceptual responses. Given the interactive nature of immersive technology, devices that capture data related to physiological states have to be wearable and not bulky or restraining while engaging in virtual reality. The field of cybersickness also requires more understanding, both on the mechanisms of how it originates and on the biomarkers through which this event can be either predicted or detected. One potential avenue of machine learning exploration could be to pair cybersickness along with other correlated or anticorrelated psychological aspects, such as vection [78] and presence [79], respectively.

Future studies could collect data from eyetrackers embedded in VR devices to track both gaze and fixation and fuse this with other already known biosignals [80]. Further on, building algorithms designed to process multiple signals in combination and independently would be especially useful in operational environments. If one source of data is cut off, another could take its place. This would maintain robust data streams for the monitorization of physiological states.

The next step in machine learning would be to generate new information every time new sample data is added (incremental, online learning) and to better model and explain related spatio-temporal brain processes. One way to do this would be to use a 3D evolving spiking neural network architecture [81, 82]. Considered the 3rd generation of artificial neural networks (ANN), spiking neural networks use spike information representation to account for changes in brain data and to learn spatio-temporal patterns from the data, which patterns can then be interpreted to understand the dynamics of the brain under certain conditions. This is in contrast to the 2nd generation ANNs, some of them reviewed above, which are not biologically plausible and do not reflect how neurons in the brain actually work in time and space under different conditions. ANNs are more computationally and energy intensive and less efficient in the interpretation of results. Perhaps, the biggest pitfall of deep neural network (DNN)-type algorithms is that they are ‘black boxes’ with many hidden layers, meaning that data interpretation is limited [83]. A dynamic evolving spiking neural network is based on the concept of an evolving connectionist system [82]. It is a modular system that evolves both structure and functionality from incoming data, in a way that is continuous, self-organized, online adaptive and interactive [84]. This makes it possible to learn spatio-temporal brain data (STBD), and the actual machine learning architecture as an interpretable model of the brain. This is useful, because unlike DNNs, it allows researchers to then act on the modelling results in a meaningful way. Architectures like NeuCube are robust to noise and create an approximate map of structural and functional cortical areas of interest using EEG data [81]. The data can be used to interpret brain activity during cybersickness experiments. Open and explainable AI systems built on brain-inspired spiking neural networks would further pave the way for integrated cybersickness prevention and alleviation techniques through better neuro-physiological data modelling, biomarker discovery and deeper understanding of personalised cybersickness processes. From an industry perspective, this information will help producers of VR content understand their consumer more, and help lift barriers to non-contact training simulations in professional fields, gaming and the building of interactive digital economies. Finally, training for resistance to cybersickness based on both objective physiological data and subjective feedback will pave the way for human adaptation to an era of ever-increasing virtual environments.

## Conclusions

This review of machine learning approaches in cybersickness studies demonstrated that a wide range of biometric and neuro-physiological signals for cybersickness identification have been analysed and discovered through the use of machine learning. Multiple machine learning architectures, modes of stimulus, VR content, environment, and participant activity have been used in studies for the automatic detection of cybersickness and prediction of further cybersick events based on these bio-markers. The predictive capabilities of current machine learning models for novel, first-instance cybersickness events, however, are still in question. Common important features have been highlighted that may be used as an input for future machine learning research in the field of cybersickness. Future research is pointed towards the collection of quality data, and the use of brain-inspired spiking neural network models [82] to achieve better accuracy and understanding of complex spatio-temporal brain processes related to cybersickness.

## Data Availability

Not applicable.

## Appendix

See Table 10.

**Table 10:** Risk of bias (ROB) assessment for 26 development and validation studies judged based on the 20 signaling question items from PROBAST [41]

Item	Paper code																									
	1	2	3	4	5	6	7	8	9	10	11	12	13	14	15	16	17	18	19	20	21	22	23	24	25	26
*Participants and data sources*																										
1	Y	Y	Y	Y	Y	Y	Y	Y	Y	Y	Y	Y	Y	Y	Y	Y	Y	Y	Y	Y	Y	Y	Y	Y	Y	Y
2	Y	N	NI	NI	Y	Y	Y	Y	Y	PN	N	N	Y	Y	N	N	Y	Y	N	Y	PY	PY	PY	Y	PN	NI
*Definition and measurement of features used for classification*																										
3	Y	Y	Y	Y	Y	N	N	Y	Y	Y	Y	Y	Y	Y	Y	Y	Y	Y	Y	Y	Y	Y	Y	Y	Y	Y
4	Y	Y	Y	Y	Y	Y	Y	Y	Y	Y	Y	Y	Y	Y	Y	Y	Y	Y	Y	Y	Y	Y	Y	Y	Y	Y
5	Y	Y	Y	Y	Y	N	N	Y	Y	Y	Y	Y	Y	Y	Y	Y	Y	Y	Y	Y	Y	Y	Y	Y	Y	Y
*How and when the outcome of cybersickness was defined and determined*																										
6	Y	Y	Y	Y	Y	Y	Y	Y	Y	Y	Y	Y	Y	Y	Y	Y	Y	Y	Y	Y	Y	Y	Y	Y	Y	Y
7	Y	Y	Y	Y	Y	Y	Y	Y	Y	Y	Y	Y	Y	Y	Y	Y	Y	Y	Y	Y	Y	Y	Y	Y	Y	Y
8	Y	Y	Y	Y	Y	Y	Y	Y	Y	Y	Y	Y	Y	Y	Y	Y	Y	Y	Y	Y	Y	Y	Y	Y	Y	Y
9	Y	Y	Y	Y	Y	Y	Y	Y	Y	Y	Y	Y	Y	Y	Y	Y	Y	Y	Y	Y	Y	Y	Y	Y	Y	Y
10	Y	Y	Y	Y	Y	Y	Y	Y	Y	Y	Y	Y	Y	Y	Y	Y	Y	Y	Y	Y	Y	Y	Y	Y	Y	Y
11	Y	Y	Y	Y	Y	Y	Y	Y	Y	Y	Y	Y	Y	Y	Y	Y	Y	Y	Y	Y	Y	Y	Y	Y	Y	Y
*Statistical methods to develop and validate the model*																										
12	PN	N	N	N	N	N	N	N	N	N	N	N	N	N	Y	N	N	Y	Y	Y	N	N	N	Y	N	N
13	Y	Y	Y	Y	Y	Y	Y	Y	Y	Y	Y	Y	Y	Y	Y	Y	Y	Y	Y	Y	Y	Y	Y	Y	Y	Y
14	N	Y	Y	Y	Y	Y	Y	Y	Y	Y	Y	Y	Y	Y	Y	Y	Y	Y	Y	Y	Y	Y	Y	Y	Y	Y
15	NI	Y	Y	Y	Y	Y	Y	Y	Y	Y	Y	Y	Y	Y	Y	Y	Y	Y	Y	Y	Y	Y	Y	Y	Y	Y
16	Y	Y	Y	Y	Y	Y	Y	Y	N	Y	Y	Y	Y	Y	Y	Y	Y	Y	Y	Y	Y	Y	Y	Y	Y	Y
17	PY	Y	Y	Y	Y	Y	Y	Y	Y	Y	Y	Y	Y	Y	Y	Y	Y	Y	N	N	Y	Y	Y	Y	Y	Y
18	PY	Y	Y	Y	Y	Y	Y	Y	Y	Y	Y	Y	Y	Y	Y	Y	Y	Y	Y	Y	Y	Y	Y	Y	Y	Y
19	NI	PY	Y	Y	Y	Y	Y	Y	Y	Y	Y	Y	Y	Y	Y	Y	Y	Y	Y	Y	Y	Y	Y	Y	Y	Y
20	Y	Y	Y	Y	Y	Y	Y	Y	Y	Y	Y	Y	Y	Y	Y	Y	Y	Y	Y	Y	Y	Y	Y	Y	Y	Y
Overall ROB	low	low	low	low	low	low	low	low	low	low	low	low	low	low	low	low	low	low	low	low	low	low	low	low	low	low

Papers are coded 1–26 from earliest to latest publication in the following order: 1. Nam et al. [12], 2. Yu et al. [13], 3. Wei et al. [14], 4. Wei et al. [16], 5. Ko et al. [15], 6. Lin et al. [17], 7. Ko et al. [18], 8. Lin et al. [19], 9. Dennison et al. [29], 10. Pane et al. [26], 11. Mawalid et al. [21], 12. Khoirunnisaa et al. [20], 13. Dennison et al. [25], 14. Wang et al. [34], 15. Garcia-Agundez et al. [28], 16. Jeong et al. [22], 17. Li et al. [35], 18. Kim et al. [42], 19. Liao et al. [27], 20. Li et al. [23], 21. Lee and Alamaniotis [43], 22. Islam et al. [30], 23. Islam et al. [31], 24. Martin et al. [33], 25. Recenti et al. [24], 26. Oh and Kim [32]

Y = yes, PY = probably yes, N = no, PN = probably no, NI = no information

PROBAST 20 signalling question items:

Selection of participants and data sources used.

1. Were appropriate data sources used?
2. Were all inclusion/exclusions appropriate?

Definition and measurement of features used for classification.

3. Were predictors defined and assessed in a similar way for all participants?
4. Were predictor assessments made without knowledge of outcome data?
5. Are all predictors available at the time the model is intended to be used?

How and when the outcome of cybersickness was defined and determined.

6. Was the outcome determined appropriately?
7. Was a pre-specified or standard outcome definition used?
8. Were predictors excluded from the outcome definition?
9. Was the outcome defined and determined in a similar way for all participants?
10. Was the outcome determined without knowledge of predictor information?
11. Was the time interval between predictor assessment and outcome determined appropriate?

Statistical methods to develop and validate the model.

12. Were there a reasonable number of participants with the outcome?
13. Were continuous and categorical predictors handled appropriately?
14. Were all enrolled participants included in the analysis?
15. Were participants with missing data handled appropriately?
16. Was selection of predictors based on univariable analysis avoided?
17. Were complexities in the data accounted for appropriately?
18. Were relevant model performance measures evaluated appropriately?
19. Were model overfitting and optimism in model performance accounted for?
20. Do predictors and their assigned weights reported in the final model correspond to the rest of the analysis?
